# Retraction: Analysis of survival for lung cancer resections cases with fuzzy and soft set theory in surgical decision making

**DOI:** 10.1371/journal.pone.0347910

**Published:** 2026-04-24

**Authors:** The PLOS One Editors

The *PLOS One* Editors retract this article [1] because it was identified as one of a series of submissions for which we have concerns about compromised peer review.

The Editors have no evidence of any author involvement in the peer review concerns.

JCRA, GV, and MFJ did not agree with the retraction. BSB and GSG either did not respond directly or could not be reached.
